# Collateral Status Evaluation Using CT Angiography and Perfusion Source Images in Acute Stroke Patients

**DOI:** 10.3390/brainsci15101092

**Published:** 2025-10-09

**Authors:** Heitor C. B. R. Alves, Bruna G. Dutra, Vivian Gagliardi, Rubens J. Gagliardi, Felipe T. Pacheco, Antonio C. M. Maia, Antônio J. da Rocha

**Affiliations:** 1 Department of Radiology and Oncology, Universidade de São Paulo, São Paulo 05508-060, SP, Brazil; bruna.dutra@hc.fm.usp.br; 2 Fleury Group, São Paulo 04344-070, SP, Brazil; 3 Irmandade Santa Casa de Misericórdia de São Paulo, São Paulo 01221-020, SP, Brazilrubensjg@apm.org.br (R.J.G.);; 4 DASA Group, São Paulo 06455-010, SP, Brazil

**Keywords:** ischemic stroke, collateral circulation, CT perfusion

## Abstract

Background/Objectives: Single-phase CT angiography (sCTA) is widely used to assess collateral circulation in acute ischemic stroke, but its static nature can lead to an underestimation of collateral flow. Our study aimed to develop and validate a direct, qualitative dynamic CTA (dCTA) collateral score based on CTP source images, without the need for post-processing software, to provide a more accurate prognostic tool. Methods: We retrospectively analyzed 112 patients with anterior circulation ischemic stroke from a prospective registry who underwent non-contrast CT, sCTA, and CTP within 8 h of onset. Collateral circulation was graded using a 4-point sCTA score and our novel 4-point dCTA score, which incorporates temporal filling patterns. We used linear regression to compare the association of both scores with CTP-derived core/hypoperfusion volumes, infarct growth, and final infarct volume. Results: The dCTA method frequently reclassified patients with poor collaterals on sCTA to good collaterals on dCTA (*n* = 23), while the reverse was rare (*n* = 5). A better collateral score was significantly associated with smaller core volume for both sCTA and dCTA, but the dCTA score demonstrated a superior model fit (R^2^ = 0.36 vs. 0.32). Similar superior correlations for dCTA were observed for hypoperfusion, infarct growth, and final infarct volumes. Critically, only the dCTA score significantly modified the association between core volume and time since stroke onset (p for interaction = 0.04). Conclusions: A collateral score derived from CTP source images (dCTA) offers a more reliable prediction of infarct lesion sizes and progression than conventional sCTA. By incorporating temporal resolution without requiring extra software, dCTA provides a robust correlation with stroke temporal evolution and represents a readily implementable tool to enhance patient selection in acute stroke.

## 1. Introduction

Recent randomized clinical trials have underscored the benefits of endovascular treatment up to 24 h post-stroke onset, leading to significant shifts in acute ischemic stroke treatment over the past decade [1,2]. However, the diverse selection criteria used in these studies have raised questions regarding the optimal imaging approach, including the role of advanced techniques like computed tomography perfusion (CTP), and whether thrombectomy could benefit a broader patient population [3,4,5]. In this context, evaluating collateral status on computed tomography angiography (CTA) has emerged as a crucial approach for patient selection, particularly in low-resource centers where access to advanced software can be limited [6,7,8].

The status of collateral circulation is a powerful predictor of infarct growth, final lesion size, and patient outcome [9,10,11,12,13,14]. Despite its importance, a significant challenge in the field has been the proliferation of different methods and grading systems for its assessment [15,16]. Single-phase CTA (sCTA) is the most used technique, evaluating collateral status by measuring the filling of distal vessels [8,17]. While simple and fast, the static nature of sCTA is a key limitation, as it lacks the temporal resolution to fully capture vessel dynamics. In contrast, multiphase CTA and dynamic CTP data provide more comprehensive information on the extent and speed of collateral filling, leading to better predictions of clinical outcome and infarct volume [18,19,20]. However, these advanced methods often require dedicated post-processing software to extract quantitative collateral maps, which has limited their widespread adoption [9,21,22].

This creates a critical gap in clinical practice: a need for a collateral scoring system that combines the temporal advantages of dynamic imaging with the practical simplicity of sCTA. As CTP becomes more mainstream following the DAWN and DEFUSE-3 trials [9,23,24,25], its role has expanded beyond patient selection. Perfusion parameters are increasingly recognized for their ability to predict not only final infarct volume but also other crucial outcomes, such as the risk of subsequent hemorrhagic transformation. This highlights the richness of CTP data, which is often underutilized [26]. We hypothesized that its source images could be leveraged for a direct, fast, and qualitative dynamic CTA (dCTA) score without needing extra software. Our aim was therefore to develop this dCTA score and compare its association with CT perfusion maps, infarct volumes, and infarct temporal evolution against the traditional sCTA score. Our findings indicate that the dCTA score offers a more robust prediction of stroke progression, providing a readily accessible tool to refine patient selection.

## 2. Materials and Methods

### 2.1. Study Design and Patient Population

This study was a retrospective analysis of a prospective institutional registry at Santa Casa de São Paulo. The registry includes all patients suspected of having an ischemic stroke between July 2011 and December 2018. The study protocol was approved by the local ethics board (Protocol: 533111116.7.0000.5479).

We included patients aged 18 years or older with an anterior circulation ischemic stroke who underwent CTA and CTP within the first 8 h of symptom onset. Exclusion criteria were: unavailable baseline clinical data, motion artifacts or excessive noise on imaging, poor contrast opacification, isolated anterior cerebral artery occlusion, bilateral or isolated cervical occlusions, and the absence of a thrombus on sCTA.

### 2.2. Image Acquisition and Processing

All patients underwent a standardized imaging protocol on a 64-channel multislice CT scanner ((Brilliance CT 64 Channel, Philips Medical, Eindhoven, The Netherlands)). The protocol consisted of a thin-slice (≤2.5 mm) baseline non-contrast computed tomography, followed by sCTA and CTP, all acquired within a 10 min window. CTP imaging was performed after the sCTA with a z-axis coverage of 4 cm. CTP images were post-processed using Olea Sphere^®^ software, version 3.0 (Olea Medical Solutions, La Ciotat, France) with a Bayesian deconvolution method. A follow-up non-contrast CT was typically performed within 24 to 72 h if the patient’s condition was stable.

### 2.3. Imaging Analyses

All imaging analyses were performed independently by two neuroradiologists (B.D. and H.A., each with eight years of stroke research experience), who were blinded to all clinical data.

The collateral score on sCTA was graded on a 4-point scale [14,17]: score 0 indicated absent collateral supply; score 1, filling > 0% but <50%; score 2, filling > 50% but <100%; and score 3, 100% collateral supply.

The novel dCTA collateral score was evaluated on CTP source images by assessing the progression of collateral filling on 20 mm axial maximum intensity projections. This was graded on a 4-point subjective scale adapted from van den Wijngaard et al. [19]: score 0 indicated poor collateral extension and slow filling; score 1, poor extension and fast filling; score 2, good extension but slow filling; and score 3, good extension and fast filling. Representative video examples of each dCTA score are provided in the Online Supplementary Materials. For analysis, a “good” collateral status on both sCTA and dCTA was defined as scores 2 or 3, while “poor” status included scores 0 and 1.

The ischemic core was defined as a relative cerebral blood flow of <40% and a time-to-maximum (Tmax) of >2 s. Hypoperfused tissue was defined as Tmax > 6 s [27]. The final infarct volume was measured manually on the first follow-up scan. Infarct growth was the difference between the final infarct and the initial core volume. The acute infarct growth rate was the core volume divided by the time from symptom onset to imaging. The clot burden score was assessed on sCTA as described by Puetz et al. [28].

### 2.4. Statistical Analyses

Patient baseline characteristics were compared between good and poor collateral groups (for both sCTA and dCTA) using chi-squared, ANOVA, or Kruskal–Wallis tests as appropriate. Interrater agreement for the collateral scores was calculated using weighted Cohen’s kappa coefficients.

Univariable and multivariable linear regression models were used to assess the associations between sCTA and dCTA scores and the primary outcomes. The multivariable models were adjusted for baseline NIHSS and clot burden score, as these were key clinical and imaging variables that were significantly different between collateral groups at baseline and are known predictors of stroke outcome. To explore the interrelationships between all parameters, a correlation matrix was generated for key clinical and imaging variables. The results were visualized as a heatmap with variables reordered by hierarchical clustering. Multiplicative interaction terms were used to test whether collateral status (good vs. poor) modified the evolution of infarct size over time. All statistical analyses were performed using R software (version 4.1.2, R Foundation for Statistical Computing, Vienna, Austria).

### 2.5. Generative AI Disclosure

The authors declare that no generative artificial intelligence (GenAI) was used for the generation of text, data, or graphics, nor for assistance in study design, data collection, analysis, or interpretation in this paper.

## 3. Results

### 3.1. Patient Population

From an initial registry of 502 patients with suspected ischemic stroke, 155 met the initial inclusion criteria. After excluding 43 patients due to factors such as unavailable data (n = 18), imaging artifacts (n = 12), or specific occlusion types (n = 13), the final analysis cohort comprised 112 patients with confirmed anterior circulation ischemic stroke.

As detailed in Table 1, patients with good collateral status on either sCTA or dCTA generally had more favorable clinical and imaging profiles, including lower baseline and 24 h NIHSS, better ASPECTS, more distal occlusions, and a smaller thrombus burden.

### 3.2. Comparison and Reliability of sCTA and dCTA Scores

**Figure 1:**
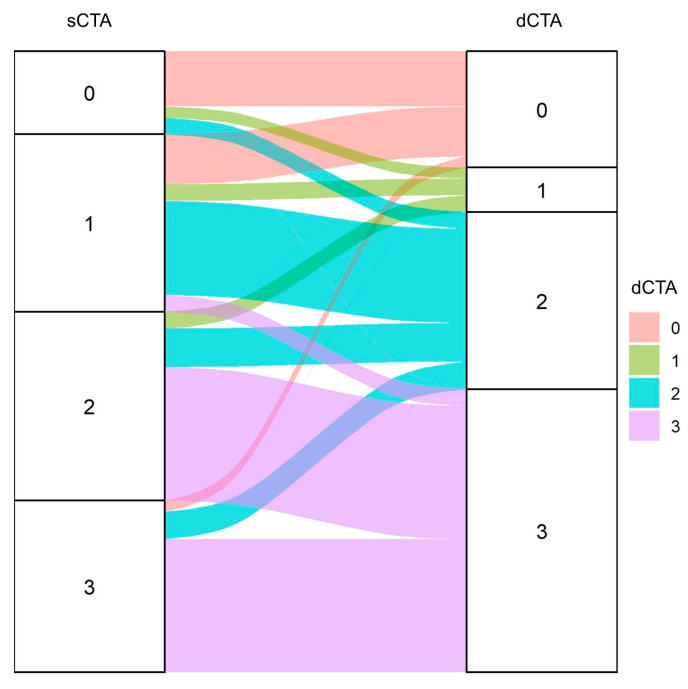
Alluvial plot illustrates the distribution and shifts in collateral scores between sCTA and dCTA. The width of each band represents the proportion of patients with a given dCTA score.

**Figure 2:**
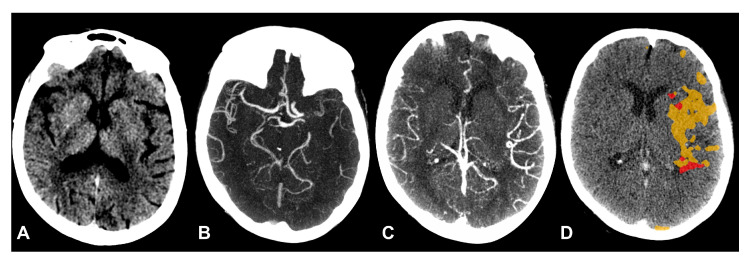
Patient with aphasia and right hemiparesis, last seen well two hours ago. There is subtle hypoattenuation on left lentiform nucleus (**A**). On CTA (**B**), there was poor filling of distal branches in MCA territory (sCTA 1). The venous phase extracted from CTP source-images (**C**) demonstrates good and delayed collateral filling (dCTA 2). The patient had a small core (red) and large penumbra (orange) volumes on CTP (**D**).

We observed a significant reclassification of collateral status between the two methods (Table 2; Figure 1). The most common shift was an upgrade from poor collaterals on sCTA to good collaterals on dCTA. For instance, 24 patients with a score of 2 on sCTA were upgraded to a score of 3 on dCTA. Furthermore, many patients initially classified as having poor collaterals on sCTA were found to have good but slow-filling collaterals, resulting in a dCTA score of 2 (Figure 2). The interrater agreement was strong and comparable for both scoring systems, with a weighted kappa statistic of 0.76 for sCTA and 0.80 for dCTA.

### 3.3. Association of Collateral Scores with Imaging Outcomes

**Figure 3:**
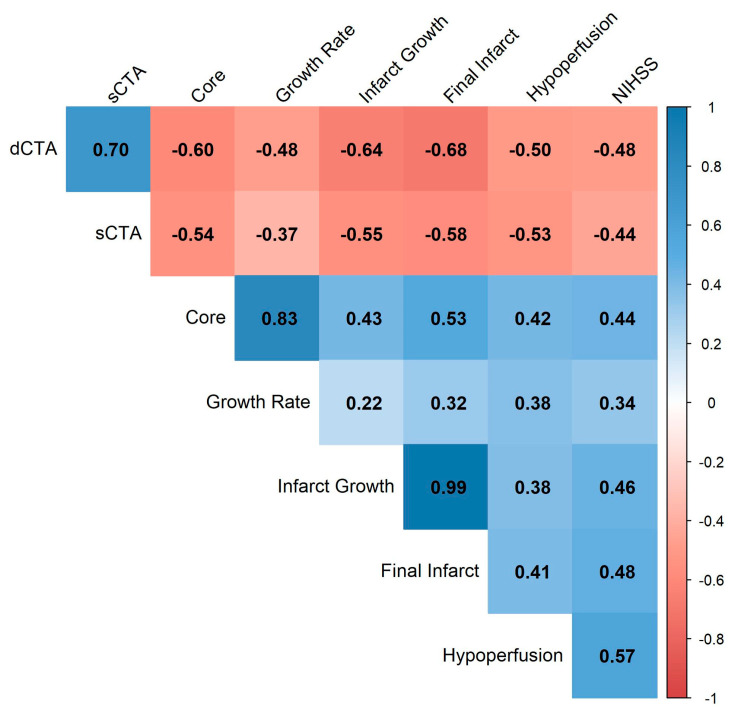
Correlation Heatmap of Stroke Parameters. The heatmap illustrates the Pearson correlation coefficients between variables. The strength and direction of the correlation are indicated by the color and the number within each square. Positive correlations are shown in blue, while negative correlations are shown in red. The intensity of the color corresponds to the strength of the correlation, as indicated by the scale bar. Variables have been reordered using hierarchical clustering to group parameters with similar correlation patterns. Abbreviations: dCTA, dynamic computed tomography angiography; sCTA, single-phase computed tomography angiography; NIHSS, National Institutes of Health Stroke Scale.

As detailed in Table 3, patients with good collateral status on both sCTA and dCTA had significantly smaller median core, hypoperfusion, infarct growth, and final infarct volumes compared to patients with poor collaterals. In the univariable linear regression analysis, a better collateral score on both sCTA and dCTA was significantly associated with all imaging outcomes (Table 4). After adjusting for baseline NIHSS and clot burden score, the prognostic value of the dCTA score remained robust, showing a significant association with smaller infarct growth (β = −48.88), final infarct volume (β = −49.29), and acute growth rate (β = −6.59). In contrast, the predictive value of the sCTA score was attenuated after adjustment, becoming non-significant for infarct growth volume. To further explore the relationships, we performed a correlation analysis, presented as a heatmap in Figure 3. The analysis confirmed strong negative correlations between better collateral scores and measures of infarct size. Notably, the dCTA score demonstrated consistently stronger negative correlations with core volume (−0.60), infarct growth (−0.64), and final infarct volume (−0.68) compared to the sCTA score (−0.54, −0.55, and −0.58, respectively).

### 3.4. Impact of Collaterals on Infarct Temporal Evolution

**Figure 4:**
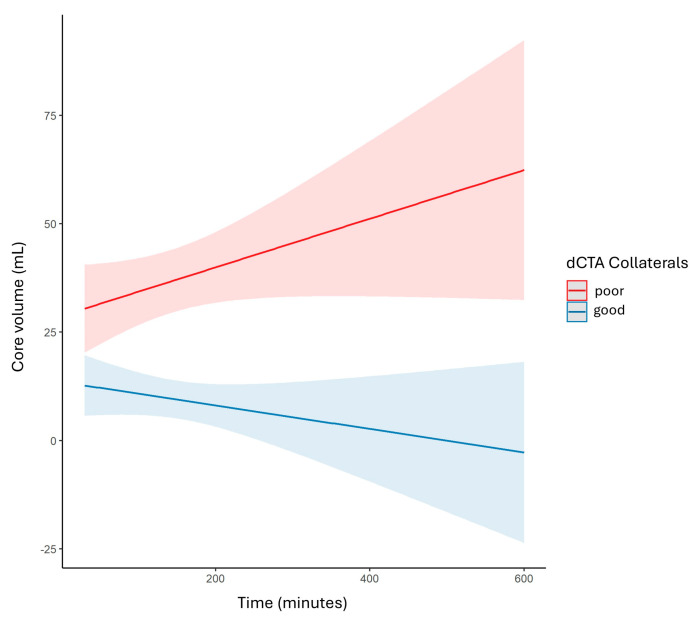
Linear regression plot illustrates significant interactions between collateral status and time on infarct core volume. Patients with poor collaterals (shown in red) exhibit a faster increase in infarct volume than patients with good collaterals (shown in blue).

The most significant difference between the two scores was their relationship with stroke progression over time. The collateral status as defined by dCTA significantly modified the association between time since stroke onset and the resulting core volume (*p*-value for interaction = 0.04). As illustrated in Figure 4, patients with poor dCTA collaterals exhibited rapid core volume progression, whereas those with good dCTA collaterals had a smaller and more stable core over time. In contrast, the sCTA score did not significantly modify this association (*p*-value for interaction = 0.32). Neither collateral score significantly modified the association between time and hypoperfusion volume.

## 4. Discussion

In this study, we demonstrate that a direct, qualitative collateral score derived from CTP source images (dCTA) is a more reliable predictor of infarct lesion size and progression than conventional sCTA. This conclusion is supported by three key lines of evidence from our analysis. First, in multivariable regression models, the dCTA score remained a strong, independent predictor of final infarct volume and infarct growth after adjusting for initial stroke severity (NIHSS) and clot burden score, whereas the predictive value of sCTA was attenuated. Second, this superior predictive capacity was visually supported by our correlation analysis, which demonstrated consistently stronger negative correlations between the dCTA score and adverse outcomes like core volume, infarct growth, and final infarct volume. Finally, our most significant finding was that only dCTA status (good vs. poor) fundamentally altered the relationship between time since symptom onset and ischemic core volume, providing a more robust link to the underlying pathophysiology of stroke evolution.

A key reason for dCTA’s superior performance is its incorporation of a temporal dimension, which helps overcome a major limitation of sCTA: the underestimation of collateral flow [29]. Our results confirm this, showing that many patients classified as having poor collaterals on a static sCTA image were reclassified as having good, albeit delayed, collaterals on dCTA. This aligns with previous work demonstrating that dynamic imaging, either through multiphase CTA or dynamic CTA, better predicts clinical outcomes and infarct volumes [18,19]. Our dCTA method is conceptually based on the work by Van den Wijngaard et al. [19], but it innovates by leveraging CTP source images directly, a novel approach.

Our general finding that better collaterals are associated with smaller core volumes is consistent with data from major trials, such as the analysis by Vagal et al. from the IMS III trial [30]. However, the literature on the relationship between sCTA collaterals and perfusion-defined penumbra has been inconsistent, highlighting the complexity of the field and the need for more refined assessment tools [31,32,33,34,35,36]. By showing a stronger and more consistent correlation with core, hypoperfusion, and final infarct volumes, our dCTA score provides a more stable and reliable assessment. This improved accuracy has significant clinical implications. For example, trials like RESILIENT exclusively enrolled patients with good collaterals [6]. Our data suggests that a dynamic assessment could identify more patients with functional, slow-filling collaterals who might otherwise be excluded from treatment based on a misleading sCTA.

The primary contribution of this work is the introduction of a simple, accessible method to improve collateral assessment. To our knowledge, this is the first study to show that a collateral score can significantly modify the relationship between core volume and time, reinforcing its validity. Crucially, it is also the first collateral score derived from CTP that does not require any post-processing software. This feature dramatically increases its applicability, especially in centers that now routinely perform both CTA and CTP. Our findings bolster the argument that when both scans are available, collateral status should be evaluated on the dynamic CTP source images.

This study has several limitations. Firstly, it is a single-center, retrospective analysis with a relatively modest sample size. Secondly, our clinical outcome data was limited, particularly regarding the 90-day modified Rankin Scale. Thirdly, the study was conducted within the Brazilian public healthcare system, where rates of endovascular treatment were low; however, this may also enhance the reproducibility of our findings in similar healthcare settings in developing countries. Finally, the 4 cm z-axis coverage of our CTP protocol may have underestimated the total ischemic volumes, though care was taken to center the slab on the affected territory. Future prospective, multi-center studies are needed to validate the dCTA score and its impact on clinical outcomes.

## 5. Conclusions

Our study demonstrates that a collateral score derived from CTP source images (dCTA) offers a more reliable prediction of infarct lesion sizes compared to conventional sCTA. By significantly modifying the association between core volume and time since stroke onset, the dCTA score provides a more robust correlation with the temporal evolution of stroke. As a direct, qualitative method that requires no additional software, dCTA is a readily implementable tool that can enhance the accuracy of imaging-based patient selection, contributing to a better understanding of stroke progression and informing future treatment strategies.

## Figures and Tables

**Table 1:** Baseline characteristics. Group comparisons were evaluated using Chi-squared, Kruskal–Wallis and student *t* tests.

	Single-Phase CTA Collaterals			CTP Source Images Collaterals		
	Poor	Good	*p* Value	Poor	Good	*p* Value
Number (n)	47	65		29	83	
Age, median [IQR]	64 [56, 77]	62 [51, 73]	0.26	58 [51, 71]	64 [56, 76]	0.12
Sex, male (%)	27 (57.4)	32 (49.2)	0.50	19 (65.5)	40 (48.2)	0.16
Intravenous alteplase, n (%)	13 (27.7)	17 (26.2)	1.00	7 (24.1)	23 (27.7)	0.90
Endovascular treatment, n (%)	1 (2.1)	1 (1.5)	1.00	0 (0.0)	2 (2.4)	0.98
Ictus in minutes, median [IQR]	120 [67, 180]	120 [60, 210]	0.64	120 [60, 180]	135 [72, 210]	0.57
NIHSS, median [IQR]	15 [13, 17]	9 [6, 14]	<0.001	15 [13, 16]	12 [6, 15]	0.01
24 h NIHSS, median [IQR]	12 [7, 14]	5 [3, 9]	<0.001	13 [10, 14]	5 [2, 10]	<0.001
SBP—mean mm Hg (SD)	150 [134, 180]	150 [130, 160]	0.11	148 [120, 200]	150 [135, 166]	0.93
DBP—mean mm Hg (SD)	90 [80, 100]	90 [80, 100]	0.19	90 [77, 100]	90 [80, 100]	0.97
Baseline glucose, median [IQR]	143 [123, 202]	120 [99, 147]	<0.001	143 [121, 213]	126 [104, 162]	0.074
Medical history						
Atrial fibrillation—n. (%)	14 (30.4)	10 (16.4)	0.17	6 (21.4)	18 (22.8)	1.00
Diabetes mellitus—n. (%)	13 (28.3)	12 (19.7)	0.42	9 (32.1)	16 (20.3)	0.31
Hypertension—n. (%)	35 (76.1)	35 (57.4)	0.07	18 (64.3)	52 (65.8)	1.00
Dyslipidemia, n. (%)	3 (6.5)	4 (6.6)	1.00	0 (0.0)	7 (8.9)	0.236
Ischemic stroke—n. (%)	4 (8.7)	7 (11.5)	0.88	2 (7.1)	9 (11.4)	0.78
Myocardial infarction—n. (%)	5 (10.9)	5 (8.2)	0.89	3 (10.7)	7 (8.9)	1.00
Peripheral artery disease—n. (%)	2 (4.3)	2 (3.3)	1.00	3 (10.7)	1 (1.3)	0.09
Imaging						
Occlusion level on CTA, n (%)			<0.001			<0.001
ICA-I	3 (6.4)	4 (6.2)		2 (6.9)	5 (6.0)	
ICA-T	17 (36.2)	5 (7.7)		12 (41.4)	10 (12.0)	
M1 proximal	13 (27.7)	7 (10.8)		7 (24.1)	13 (15.7)	
M1 distal	9 (19.1)	12 (18.5)		3 (10.3)	18 (21.7)	
M2	5 (10.6)	18 (27.7)		5 (17.2)	18 (21.7)	
M3	0 (0.0)	16 (24.6)		0 (0.0)	16 (19.3)	
M4	0 (0.0)	3 (4.6)		0 (0.0)	3 (3.6)	
Clot Burden Score, median [IQR]	4 [2, 6]	9 [6, 10]	<0.001	4 [2, 6]	7 [5, 9]	<0.001
Hyperdense artery sign, n. (%)	38 (80.9)	25 (39.1)	<0.001	22 (75.9)	41 (50.0)	0.03
Thrombus length in mm, median [IQR]	18.25 [13.32, 24.63]	9.09 [4.36, 12.90]	<0.001	15.40 [10.84, 22.50]	11.73 [5.52, 17.93]	0.02
Tandem occlusion, n. (%)	5 (11.6)	2 (3.3)	0.20	5 (18.5)	2 (2.6)	0.01

Abbreviations:ASPECTS indicates Alberta Stroke Program Early CT Score; CTA, computed tomography angiography; CTP, computed tomography perfusion; DBP, diastolic blood pressure; ICA-I, intracranial internal carotid artery; IQR, interquartile range; NIHSS, National Institutes of Health Stroke Scale; SBP, systolic blood pressure; SD, standard deviation.

**Table 2:** Dynamic computed tomography angiography (dCTA) collateral score stratified by the single-phase computed tomography angiography (sCTA).

sCTA	dCTA			
	0	1	2	3
0	10	2	3	0
1	9	3	17	3
2	0	3	7	24
3	2	0	5	24

**Table 3:** Imaging Outcomes Based on sCTA and dCTA Collateral Status.

	Single-Phase CTA Collaterals			CTP Source Images Collaterals		
	Poor	Good	*p* Value	Poor	Good	*p* Value
Number (n)	47	65		29	83	
Core volume (mL), median [IQR]	21.5 [8.8, 36.0]	3.9 [1.1, 7.6]	<0.001	27.1 [11.0, 66.8]	4.63 [1.3, 11.8]	<0.001
Hypoperfusion volume (mL), median [IQR]	113.3 [82.3, 135.7]	33.9 [13.3, 81.2]	<0.001	123.3 [83.6, 151.8]	51.4 [22.1, 99.1]	<0.001
Infarct growth volume (mL), median [IQR]	118.6 [48.4, 290.5]	24.9 [7.6, 42.9]	<0.001	182.6 [107.6, 345.4]	27.5 [11.8, 78.3]	<0.001
Final infarct volume (mL), median [IQR]	120.6 [53.1, 303.4]	25.4 [12.2, 44.9]	<0.001	176.0 [101.3, 350.1]	27.3 [13.1, 92.0]	<0.001
Acute growth rate (mL/min), median [IQR]	1.41 [0.1, 6.0]	0.0 [0.0, 0.16]	<0.001	2.3 [0.4, 13.1]	0.0 [0.0, 1.1]	<0.001

IQR, Abbreviations, interquartile range.

**Table 4:** Univariable and Multivariable Linear Regression Analyses of Collateral Scores and Imaging Outcomes.

		sCTA			dCTA		
Outcomes	Model	β (95% CI)	R-Squared	*p*-Value	β (95% CI)	R-Squared	*p*-Value
Core volume	unadjusted	−13.22 (−16.97 to −9.46)	0.32	<0.01	−12.68 (−15.92 to −9.44)	0.36	<0.01
	Adjusted *	−10.17 (−16.48 to −3.86)	0.32	<0.01	−9.08 (−14.61 to −3.55)	0.33	<0.01
Hypoperfusion volume	Unadjusted	−29.64 (−39.06 to −20.21)	0.27	<0.01	−30.96 (−38.82 to −23.09)	0.37	<0.01
	Adjusted *	−8.25 (−23.9 to 7.39)	0.42	0.29	−11.56 (−23.93 to 0.81)	0.44	0.07
Infarct growth volume	Unadjusted	−62.25 (−87.56 to −36.93)	0.24	<0.01	−65.88 (−87.22 to −44.54)	0.33	<0.01
	Adjusted *	−34.89 (−71.7 to 1.92)	0.36	0.06	−48.88 (−79.2 to −18.56)	0.43	<0.01
Final infarct volume	Unadjusted	−68.07 (−93.35 to −42.80)	0.26	<0.01	−68.38 (−89.98 to −46.78)	0.33	<0.01
	Adjusted *	−45.69 (−78.6 to −12.78)	0.40	<0.01	−49.29 (−80.25 to −18.33)	0.43	<0.01
Acute growth rate	Unadjusted	−3.73 (−6.39 to −1.06)	0.07	<0.01	−4.76 (−7.02 to −2.49)	0.15	<0.01
	Adjusted *	−5.28 (−11.46 to 0.9)	0.12	0.09	−6.59 (−11.39 to −1.79)	0.19	<0.01

* Adjusted models include baseline NIHSS and clot burden score as covariates.

## Data Availability

The datasets generated and analyzed during the current study are not publicly available because they contain sensitive clinical information from patients. Public sharing of this data would compromise patient confidentiality and violate the terms of our ethics approval. However, de-identified data may be made available from the corresponding author upon reasonable request and subject to a data sharing agreement and ethics board approval.

## Supplementary Materials

The following supporting information can be downloaded at: https://www.mdpi.com/article/10.3390/brainsci15101092/s1; Video S1: dCTA score of 0, demonstrating poor collateral extension and slow filling; Video S2: dCTA score of 1, demonstrating poor collateral extension and fast filling; Video S3: dCTA score of 2, demonstrating good collateral extension but slow filling; Video S4: dCTA score of 3, demonstrating good collateral extension and fast filling.

## Abbreviations

The following abbreviations are used in this manuscript:

AIS	acute ischemic stroke
CTP	computed tomography perfusion
dCTA	dynamic computed tomography angiography
sCTA	single-phase computed tomography angiography
